# Harnessing information and communication technologies to leverage scarce resources for cancer education, research and practice in developing countries

**DOI:** 10.1186/1478-4505-4-1

**Published:** 2006-01-03

**Authors:** Valentine B Andela

**Affiliations:** 1 Cancer-Africa Inc. Miami, Florida

## Abstract

In developing countries, low levels of awareness, cost and organizational constraints on access to specialized care contribute to inadequate patient help-seeking behavior. As much as 95% of cancer patients in developing countries are diagnosed at late to end stage disease. Consequently, treatment outcome is dismally poor and a vicious cycle sets in, with public mystification of cancer and the admonishment of cancer medicine as a futile effort, all, to the further detriment of patient help-seeking behavior and treatment engagement. The situation spirals down, when the practice of cancer medicine is not gratifying to the medical practitioner and does not appeal as a medical specialty to those in training. The future of cancer medicine in developing countries thus hinges on the demystification of cancer through positive information, coupled to an effective organization that allows for the optimal use of available resources, facilitates access to specialized care and promotes the flow of knowledge and technology amongst various stakeholders. This paper strives to make a cogent argument and highlight the capital importance of information and communication technologies in organizing and leveraging scarce resources for cancer education, research and practice in developing countries.

## Background

Developing countries currently bear fifty-percent of the global cancer burden and, are projected to bear sixty-percent by the year 2020 [1]. The rise in the cancer case-incidence is for the most part ascribed to social, lifestyle and environmental changes concurrent with economic development and globalization [2,3]. Primarily, the resources mobilized towards improving public health practices and health care delivery, have significantly improved the control capacity of acute life-threatening conditions, increased life expectancies and brought forth chronic conditions such as cancer. Contemporaneously, the HIV/AIDS burden of developing countries would go from a "plague-like" to a manageable chronic endemic condition with its associated malignant complications [4,5]. Secondarily, economic development is bringing about lifestyle changes that significantly increase the lifetime risk of developing cancer. Notable lifestyle changes include a significant uptake in tobacco and alcohol; increased consumption of processed foods low in fiber and high in refined sugars and saturated fat; increased sedentariness as a result of the mechanization of household chores, transportation and industrial activities [2,3]. Furthermore, increased industrial activity results in an increase in environmental pollutants and lifetime exposure to carcinogens [2].

In the wake of an overwhelming cancer case-incidence, an already over-extended healthcare system gives way. Presently, because of low levels of awareness and the cost and organizational constraints on access to specialized care as much as 95% of cancer patients in developing countries are diagnosed at late or end stage disease [6-8]. Consequently, treatment outcome is dismally poor [9-11]. A vicious cycle sets in, with public mystification of cancer and the admonishment of cancer medicine as a futile effort, all to the further detriment of patient help-seeking behavior and treatment engagement. The situation spirals down, when the practice of cancer medicine is not gratifying to the medical practitioner and does not appeal as a medical specialty to those in training.

By contrast, with existing knowledge and technology, the concerted action of governments, non-governmental organizations and healthcare systems in developed countries can prevent a third of cancers, provide treatment to another third and palliative care to those in need [12]. While this represents a significant stride in the "war against cancer", cancer remains the leading cause of morbidity and mortality in developed countries [1,13,14]. This mandates for sustained efforts in the areas of population / epidemiological, and clinical research, where certain limitations have emerged that must be emphasized. Primarily, the relative homogeneity of lifestyle and environmental exposure in developed countries limits the study of cancer promoting and counteracting factors [2]. Secondly, with almost daily and exponential advances in basic and translational research findings, novel cancer drugs and cancer preventive and therapeutic strategies are designed yet not tested because of a contracting patient pool available and willing to participate in clinical trials [15,16]. It is in fact estimated that more than 80% of biotechnology and pharmaceutical companies miss their deadlines for patient enrollment and loose over 1.3 million dollars in direct and opportunity cost [17]. Overarchingly, the culture of science in western societies has not wholly integrated the complementary culture of science in other societies [18]. As a matter of fact, cross-collaborations yield profound results and there is much to be gained from engaging a south-south and north-south dynamic [2,18,19].

## Bridging the chasm

Historically, the sharing advantage of nascent technologies has spurred leaps and bounds in human development. This is evident in the telescoping timelines of human development milestones, from a ten thousandth year lapse for the onset of the agricultural revolution, to a four hundredth year lapse for the scientific revolution and then a one hundredth year lapse for the industrial revolution. In order, the information revolution heralds an unsurpassed leap in national, international and global development, by granting access to knowledge, cognitive tools and organizational support and permitting a greater fraction of the global community to actualize their human potentials and participate in a global intelligent network.

By virtue of its sharing advantage (portability and affordability), developing countries have in recent times, known an excess of 263% growth in telecommunications and Internet connectivity [20,21]. The growth and penetration of telecommunications and Internet connectivity in developing countries has in fact already triggered a wide scale and successful adoption of such media in various development initiatives operating at the grass-roots level, notably in the areas of education and healthcare [22-24]. Such initiatives set precedence and chart a path for the successful implementation of the information and communication technologies (ICTs) towards enhancing cancer education, research and practice.

### Cancer education

Knowledge, positive information and advocacy are cornerstones of cancer survivorship [25,26]. In developing countries, the low level of cancer knowledge and awareness in the general population has had devastating consequences. Generally, cancer is mystified, preventative actions are not taken and treatment engagement is deficient. Mass education, positive information and advocacy should thus be a mainspring in capacity building. By raising the level of awareness, a critical mass would be attained and a dynamic intercourse would result amongst patients, providers, policy makers and the public, towards mobilizing resources and taking corrective action. The penetration of ICTs in developing countries validates their utility as a mass media with unique advantages over traditional (audio-visual) mass media. Notably, the Internet medium grant(s) access to interactive tools that generate evidence-based medical information pertinent to cancer prevention and treatment and customized to the individuals' unique characteristics [27,28]. Furthermore, the interactivity afforded by online chat-rooms, e-mails and more importantly wireless application protocols (short messages services sent to individual cell phone users), all at minimal to no cost, gives tremendous potential for a sustainable effort in individualized and customized education, advocacy and support.

### Cancer research

Information engenders research and research engenders information. To this end, the Internet would not only serve the informational and educational purposes, but would inherently result in the digitization of data, procedures and processes and set up the quintessence of a rigorous and viable research enterprise: speed, control, accountability, cost-containment and patient-provider engagement and satisfaction [29,30].

### Cancer practice

Optimal cancer patient care is multidisciplinary and at its bare minimum, calls for the timely and concerted opinion and action of the patient; the primary care physician; the pathologist; the medical oncologist; the radiation oncologist and the surgical oncologist [31]. In the setting of a developing country, multidisciplinary cancer patient care is limited by educational, cost and organizational constraints [6-11]. The uninformed patient seeks medical attention from inadequate sources and expends a great deal of resources in the process [8]. These deficiencies are compounded by the limited number of trained professionals in cancer medicine and the quasi inexistence of professional networks[3,8,9]. Again the Internet medium would not only serve the informational and educational purposes, but would inherently result in a self-organized community wherein cancer patients and care providers seamlessly interact. Harnessing the organizational support the Internet medium affords, would clearly result in speed, control, accountability, cost-containment and patient-provider engagement and satisfaction [29,30].

## Recommendation and conclusion

The strength of ICTs should be harnessed to engage a south-south and north-south dynamic. While laudable efforts are being made towards building the cancer control capacity of developing countries, by transfering technology from developed countries [32,33], the prevailing platform, that is humanitarian aid and distributive justice, is weak and potentially detrimental [34,35]. Transferring appropriate knowledge and technology from developed countries is desirable, yet should not undermine the opportunities that abound in developing countries and that should be transformed and harnessed towards improved cancer care [2,17]. It is intended that putting a spotlight on these opportunities and threats would stimulate improved and dynamic partnerships in the global fight against cancer [36].

## Disclosures

The author is a UICC fellow and member of the UICC expert review panel, focusing on the area of cancer information and education.

**Figure 1:**
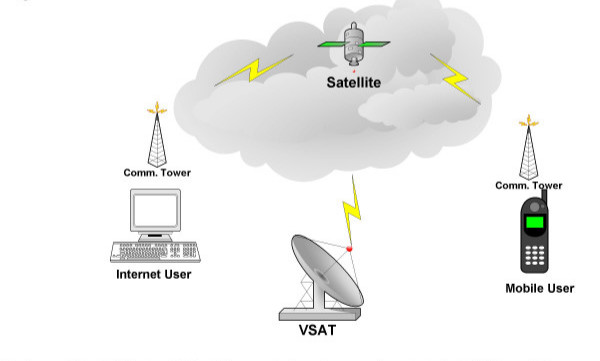
**Hardware and network architecture**: Global **satellite **communications using very small aperture terminals (**VSAT**) are widely distributed in urban and sub-urban localities, where economic transitions concur with a rise in cancer case incidences [3]. Widespread connectivity (**Internet user**) and the added potential for interactivity with the **mobile user**, all at low to minimal cost, supports the use of these media in cancer education and advocacy efforts.

**Figure 2:**
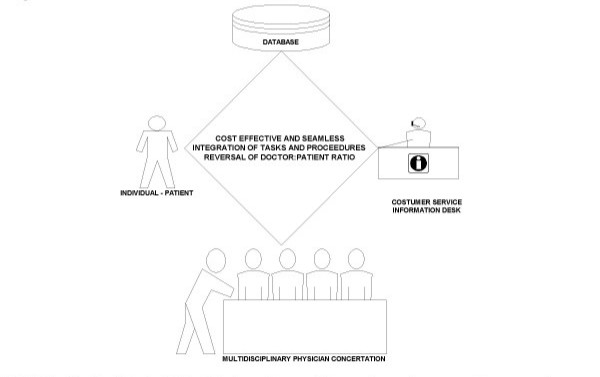
**Public-patient-physician networks**: With the digitization and storage of data, procedures and processes, a rigorous research enterprise and evidence-based medical practice would result, characterized by speed, control, accountability, cost-containment and patient-provider engagement and satisfaction.

## References

[B1] Shibuya K, Mathers CD, Boschi-Pinto C, Lopez AD, Murray CJ (2003). Global and regional estimates of cancer mortality and incidence by site: II. Results for the global burden of disease. BMC Cancer.

[B2] Rastogi T, Hildesheim A, Sinha R (2004). Opportunities for cancer epidemiology in developing countries. Nat Rev Cancer.

[B3] Walker AR (1995). Cancer outlook: an African perspective. J R Soc Med.

[B4] Orem J, Otieno MW, Remick SC (2004). AIDS associated cancer in developing nations. Curr Opin Oncol.

[B5] Chokunonga E, Levy LM, Bassett MT, Borok MZ, Mauchaza BG, Chirenje MZ, Parkin DM (1999). Aids and cancer in Africa: the evolving epidemic in Zimbabwe. AIDS.

[B6] Were EO, Buziba NG (2001). Presentation and health care seeking behaviour of patients with cervical cancer seen at Moi Teaching and Referral Hospital, Eldoret, Kenya. East African Medical Journal.

[B7] Loehrer PJ, Greger HA, Weinberger M Knowledge and beliefs about cancer in a socioeconomically disadvantaged population. Cancer.

[B8] Yomi J, Gonsu FJ (1995). Social, economical and educational causes of late diagnosis and treatment of cancer in Cameroon. Bull Cancer.

[B9] Thomas J (2004). Cancer control in Africa: a call for action. Afr J Med Med Sci.

[B10] Anh PT, Duc NB (2002). The situation with cancer control in Vietnam. Jpn J Clin Oncol.

[B11] Pal SK, Mittal B (2004). Fight against cancer in countries with limited resources: the post-genomic era scenario. Asian Pac J Cancer Prev.

[B12] UICC Annual Report. http://www.uicc.org.

[B13] Jemal A, Murray T, Ward E, Samuel A, Tiwari RC, Ghafoor A, Fever EJ, Thun MJ (2005). Cancer Statistics, 2005. CA Cancer J Clin.

[B14] Doll R (1989). Progress against cancer: are we winning the war?. Acta Oncol.

[B15] Lara PN, Higdon R, Lim N, Kwan K, Tanaka M, Lau DH, Wun T, Welborn J, Meyers FJ, Christensen S, O'Donnell R, Richman C, Scudder SA, Tuscano J, Gandara DR, Lam KS (2001). Prospective evaluation of cancer clinical trial accrual patterns: identifying potential barriers to enrollment. J Clin Oncol.

[B16] Grunfeld E, Zitzelsberger L, Coristine M, Aspelund F (2002). Barriers and facilitators to enrollment in cancer clinical trials: qualitative study of perspectives of clinical research associates. Cancer.

[B17] Toumou R (2004). Obstacles and challenges to conducting clinical research in developing countries. Masters Thesis Georgetown University, Clinical Research Administration Program.

[B18] Iaccarino M (2003). Western science could learn a thing or two from the way science is done in other cultures. EMBO Reports.

[B19] Ngu VA (1972). Chemotherapy of Burkitts tumor at the University of Ibadan, Nigeria. JAMA.

[B20] ITU World Telecommunication Development Report 2002. http://www.itu.int.

[B21] Global Internet geography. http://www.telegeography.com/products/gig/index.php.

[B22] International Telecommunications Union (2000). TeleMedicine And Developing Countries. Telecommunications Development Bureau, Document 2/116.

[B23] Fraser H, McGrath S (2000). Economical Solutions are Available to Support Health Care In Remote Areas. British Medical Journal.

[B24] Sustainable Development Networking Program. http://www.sdnp.undp.org.

[B25] Clark EJ, Stovall EL (1996). Advocacy: the cornerstone of cancer survivorship. Cancer Practice.

[B26] Leigh S, Clark E, Berger A, Portenoy RK, Weissman DE (1998). Psychosocial aspects of cancer survivorship. Principles and Practice of Supportive Oncology.

[B27] Kim DJ, Rockhill B, Colditz GA (2004). Validation of the Harvard Cancer Risk Index: a prediction tool for individual risk. J Clin Epidemiol.

[B28] Delvenne C, Pasleau F (2003). Organizing access to evidence-based medicine resources on the web. Compt Methods Programs Biomed.

[B29] Haigh PJ (1993). Healthcare reengineering via network technologies: a critical combination. Comput Healthc.

[B30] Flower J (2003). Transformations of the 21st century heatlh care, Part 1. Beyond the digital divide. Health Forum J.

[B31] Fried BJ, Leatt P, Deber R, Wilson E (1998). Multidisciplinary teams in health care: lessons form oncology and renal teams. Healthc Manage Forum.

[B32] Harris E (2004). Building scientific capacity in developing countries: Simply transferring knowledge and instrumentation is not enough to help developing countries build their own research base. Such efforts must be tied to national and local needs to create trust and services for society in the long term. EMBO Reports.

[B33] Ianccarino M (2004). Mastering science in the South: to develop much needed research capacity, developing countries cannot rely on the industrial world, but have to find their own solutions. EMBO Reports.

[B34] Varmus H, Satcher D (1997). Ethical complexities of conducting research in developing countries. N Engl J Med.

[B35] Lurie P, Wolfe SM (1997). Unethical trials of interventions to reduce perinatal transmission of the human immunodeficiency virus in developing countries. N Engl J Med.

[B36] Costello A, Zumla A (2000). Moving to research partnerships in developing countries. British Medical Journal.

